# Multiple constraints on urban bird communication: both abiotic and biotic noise shape songs in cities

**DOI:** 10.1093/beheco/arab058

**Published:** 2021-07-13

**Authors:** Ann W Y To, Caroline Dingle, Sarah A Collins

**Affiliations:** 1 School of Biological and Marine Sciences, Faculty of Science and Engineering, University of Plymouth, Drake Circus, Plymouth PL4 8AA, Devon, UK; 2 School of Biological Sciences, Faculty of Science, University of Hong Kong, Pokfulam Road, Hong Kong

**Keywords:** bird songs, insect noise, multiple noise sources, urban noise

## Abstract

Ambient noise can cause birds to adjust their songs to avoid masking. Most studies investigate responses to a single noise source (e.g., low-frequency traffic noise, or high-frequency insect noise). Here, we investigated the effects of both anthropogenic and insect noise on vocalizations of four common bird species in Hong Kong. Common Tailorbirds (*Orthotomus sutorius*) and Eurasian Tree Sparrows (*Passer montanus*) both sang at a higher frequency in urban areas compared to peri-urban areas. Red-whiskered Bulbuls (*Pycnonotus jocosus*) in urban areas shifted the only first note of their song upwards. Swinhoe’s White-eye (*Zosterops simplex*) vocalization changes were correlated with noise level, but did not differ between the peri-urban and urban populations. Insect noise caused the Eurasian Tree Sparrow to reduce both maximum, peak frequency, and overall bandwidth of vocalizations. Insect noise also led to a reduction in maximum frequency in Red-whiskered bulbuls. The presence of both urban noise and insect noise affected the sound of the Common Tailorbirds and Eurasian Tree Sparrows; in urban areas, they no longer increased their minimum song frequency when insect sounds were also present. These results highlight the complexity of the soundscape in urban areas. The presence of both high- and low-frequency ambient noise may make it difficult for urban birds to avoid signal masking while still maintaining their fitness in noisy cities.

## Teaser

Studies have shown that birds increase their vocalization pitch under low-frequency urban noise and sing at a lower pitch when exposed to high-frequency insect sounds, but what if they occur simultaneously? Using field recordings, we found that some urban birds no longer adjusted their song in response to urban noise when insect noise is also present. This shows that anthropogenic noise is not the only driver of changes in urban bird songs.

## Introduction

Avian acoustic signals are important for mate attraction, territorial defense, alarm signaling, and other functions vital for survival and fitness (Collins 2004, Catchpole and Slater 2008, Bradbury and Vehrencamp 2011). However, the efficient transmission of vocal signals is affected by the presence of ambient noise from both biotic and abiotic sources, potentially leading to lower fitness (Brumm and Slabbekoorn 2005; Slabbekoorn 2013, McMullen et al. 2014; Kleist et al. 2018). To avoid masking by noise, species may alter their vocalizations through shifts in frequency, amplitude, song rate, and duration of song (Cardoso and Atwell 2011; Goodwin and Podos 2013; Shannon et al. 2016; de Magalhães Tolentino et al. 2018; Lee & Park 2019; Lowry et al. 2019).

To date many species of birds have been shown to alter their vocalizations to avoid signal overlap with abiotic (e.g., rushing water, Pytte et al. 2003), or biotic factors; such as the songs of other birds (Planqué and Slabbekoorn 2008), insect sounds (Slabbekoorn and Smith 2002; Dingle et al. 2008; Kirschel et al. 2009; Luther 2009; Hart et al. 2015; Stanley et al. 2016), and amphibian choruses (Lenske and La 2014). Anthropogenic noise is a more recent phenomenon, but birds also show a wide range of acoustic responses to low-frequency anthropogenic noise in particular. The most commonly documented responses of birds to low-frequency anthropogenic noise are to increase song frequency and/or amplitude in order to avoid signal masking (e.g., Slabbekoorn and Peet 2003; Brumm 2004; Wood and Yezerinca 2006; Slabbekoorn 2013; Derryberry et al. 2016, 2017; Guo et al. 2016; Zollinger 2017). Increases in frequency and amplitude could be directly linked, with birds singing louder also singing at higher frequencies as a correlated response, rather than an independent change (Brumm and Zollinger 2011; Zollinger et al. 2012; Zollinger et al. 2012); or higher frequencies may be used by birds as these frequencies allow greater amplitude (Nemeth et al. 2013). However, several studies have shown that birds decrease the frequency of their vocalizations in response to high-frequency noise; suggesting that frequency responses can be independent of adjustments of amplitude (Great Tits, *Parus major*, Halfwerk and Slabbekoorn 2009; Black capped chickadees, *Poecile atricapillus*, Courter et al. 2020).

Signal masking due to high levels of anthropogenic noise has been linked to a reduction in mating and reproductive success, and decreases in abundance (Habib et al. 2007; Gross et al. 2010; Ríos-Chelén et al. 2013; Slabbekoorn 2013, Francis 2015; Shannon et al. 2016; Senzaki et al. 2020). Although birds may avoid some of the negative impacts of signal masking by changing the way in which they sing, such noise-dependent adjustments may impact mate attraction under anthropogenic noise (Moiron et al. 2015; Luther et al. 2016), particularly in species with lower frequency vocalizations (Francis 2015).

The majority of studies on the impact of noise on animal communication have focused on responses to a single noise source. However, in tropical urban areas, birds are frequently exposed to both low-frequency anthropogenic noise and high-frequency cicada choruses, often simultaneously. Many cicada species generate high intensity and long-lasting acoustic signals for mate attraction. While traffic noise is usually below 2 kHz, cicada calls can range from 1 to 25 kHz and can generate noise of up to 148.5 dB (Young and Bennet-Clark 1995; Fonseca et al. 2000). Birds attempting to communicate under such conditions could be impacted by signal masking both at the low and high limits of their song frequency range. Between increasing the frequencies of their songs in response to low-frequency traffic noise, and decreasing in response to high-frequency insect noise, birds could find that they are limited to a relatively narrow bandwidth for efficient communication.

In this study, we recorded vocal signals of four common urban passerines in a dense semitropical urban area, to study the effect of multiple sources of background noise on song structure. We aimed to: 1) investigate whether birds alter their vocalizations in the presence of anthropogenic or insect noise; 2) test for an interaction effect of anthropogenic noise and insect noise on song; and 3) determine whether any response observed correlated with the amplitude of anthropogenic noise or frequency of the cicada chorus. Based on previous studies, we predicted that birds would sing with higher frequencies in the presence of anthropogenic noise, lower frequencies in the presence of insect noise, and would produce songs with narrower bandwidths under conditions where both noise sources were present.

## METHODS

### Sampling location and period

This study was conducted in Hong Kong, a densely populated urban area with 8.8 million citizens living on 1106 km^2^ of land (Information Service Department 2018). Despite the high population densities in some parts of Hong Kong, approximately 40% of land within the region is designated as a country park or protected area.

**Figure 1:**
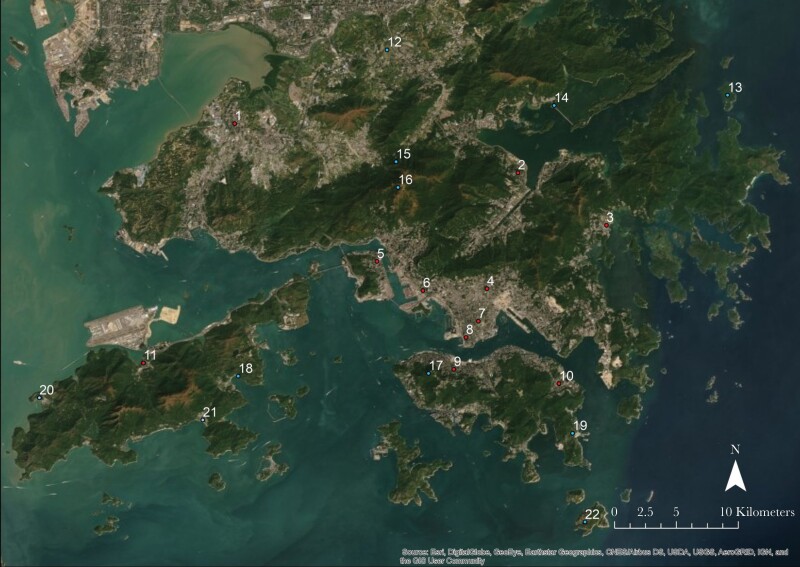
Map shown the sampling location of urban sites (red) and peri-urban sites (blue) across Hong Kong.

We collected bird songs and noise measurement data from 11 urban and 11 peri-urban sites across Hong Kong between 17 June and 8 September 2013 (Figure 1). Cicadas across Hong Kong are mainly active from early May to late September every year and can be found in both urban and rural areas, and were actively vocalizing during the whole sampling period. The weather was similar across recording days: maximum temperature was in range of 30.3–31.1 °C, minimum temperature was 25.7–26.5 °C, relative humidity was 82–85%, and rainfall was 436.3–445.4 mm (Hong Kong Observatory 2021). These sampling locations were classified as urban and peri-urban based on the nature of the surrounding buildings and habitats in the area. For example, sites with high traffic highways and high-density residential buildings were classified as urban areas, and we expected these urban areas to be noisier at low frequencies than peri-urban sites. Urban sites included urban parks (seven sites) or roadside green spaces (four sites), while peri-urban sites were in, or next to, protected areas (four sites), traditional rural villages (five sites), or outlying islands (two sites). Visits were made to each sampling location once during the study period between 0600 and 1400 local time (UTC +8:00). At each site, songs were recorded along a single transect, ranging from 1.5 to 4.2 km. Duration of recording ranged from 2 to 5 h based on the length of transects. All transects followed accessible routes throughout the sampling site, such as roads, trails, and footpaths. We recorded all birds that sang within the sampling period. The recording started as soon as a bird was heard singing, and stopped after the bird ceased singing. To avoid recording the same individual twice within the same site, recordings were made at least 25 m apart. If more than one individual of the same species was singing at the same time, all songs in that recording were analyzed, but this was counted as one sample only. Data collection occurred under fine weather conditions, i.e. no rain or strong wind. For sites on outlying islands, we did not sample near the coastline to limit the impact of low-frequency noise produced by wave action.

### Recordings and noise measurement

We recorded songs with a TASCAM DR-40 digital recorder (TASCAM, Japan) and a Superlux PRA118L shotgun microphone (Superlux, Taiwan) with windscreen. Recordings were set to mono channel mode at 24-bit WAV with 44.1 kHz sampling rate, no cut-off frequency function was applied. All birds singing along the transect line were recorded with the same settings.

The background noise level at each site was measured using a WESEN WS1361 (WESEN, China) Type II sound level meter using C-weighting due to its sensitivity to low-frequency noise. The sound level meter was set on a tripod at 1.2 m in height and at least 1 m away from any surface to avoid sound reflection, which would result in a higher reading (Environmental Protection Department 1997). This background noise level measurement was focused on the low-frequency anthropogenic noise, so the noise level measurement was paused when insect noise occurred in the environment. Sound measurements were taken in three different directions (000°, 120°, 240°), using the *L*_eq(C)_ (equivalent continuous sound level in C-weighting) measurement for 5 min each at the start and the end of sampling. We calculated overall background noise level for each site by averaging these values (Equation 1).


$$\begin{array}{l} L_{eq(C)}=10\log\underset{i=t}{\overset{i=n}{\sum }}\;\left(10^{\frac{L_{i}}{10}}\right)(\frac{1}{t_{i}}) \end{array}$$ (1)

### Study species

Of all of the species encountered along transects, we chose those with a minimum of twenty individuals recorded in both urban and peri-urban areas for further analysis. Based on such criteria, only the following four species were included in the analysis: Common Tailorbird (*Orthotomus sutorius*); Eurasian Tree Sparrow (*Passer montanus*); Swinhoe’s White-eye (*Zosterops simplex*); and Red-whiskered Bulbul (*Pycnonotus jocosus*). For these four species, a total of 272 individual birds were recorded from 22 sites. No species studied was recorded in all 22 sites during the sampling period. The range of sample size of each species per site was from 0 to 13 (Table 1). Among all the samples, 54% of urban locations and 61% of peri-urban locations had insect sounds present in the background during the recording period.

**Table 1:** Sample size, individuals, (*N*) of the four studied species. The number of sites (*N* of site) indicates how many sites the samples were collected from

	*N*	
Species	Peri-urban (*N* of site)	Urban (*N* of site)
Common Tailorbird (*Orthotomus sutorius*)	41 (9)	22 (8)
Eurasian Tree Sparrow (*Passer montanus*)	26 (6)	52 (11)
Swinhoe’s White-eye (*Zosterops japonicus*)	39 (9)	41 (10)
Red-whiskered Bulbul (*Pycnonotus jocosus*)	29 (7)	22 (9)

### Sound analysis

**Figure 2:**
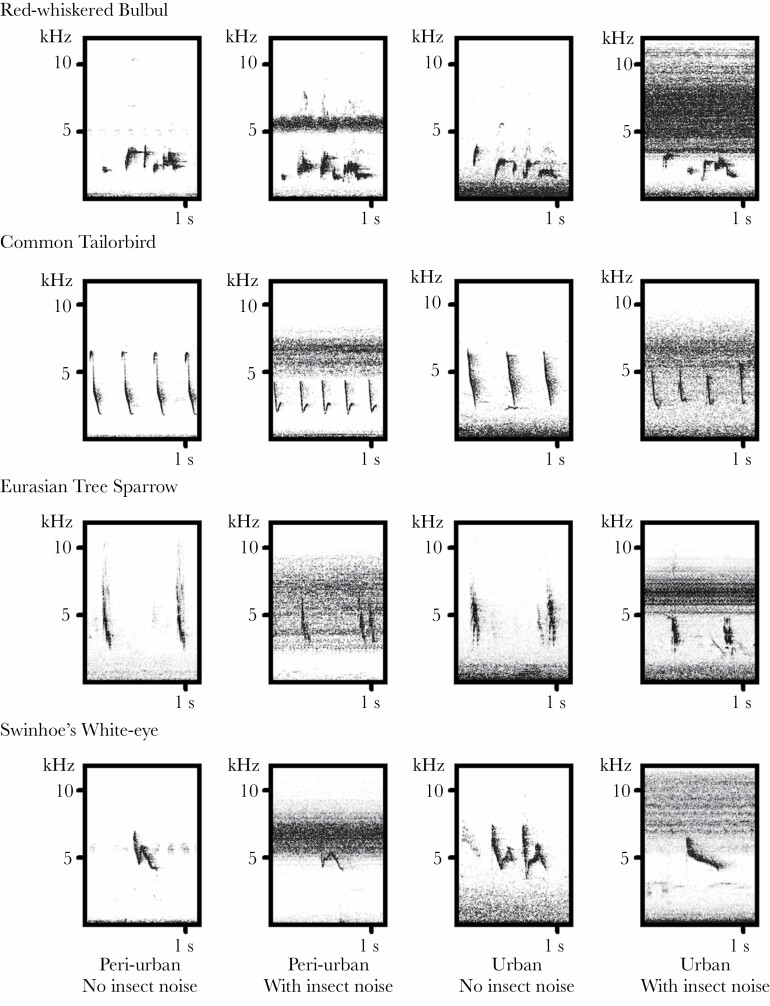
Spectrograms showed the vocalizations of the four species studied under different ambient noise situations.

As many species sing multiple types of vocalizations, for this study we chose one specific type of vocalization of each species (Figure 2) for further analysis using Avisoft SASLab Pro Version 5.2.06 (Avisoft Bioacoustic, Berlin, Germany). Spectrogram settings were the following: FFT length 1024 with 100% frame size and Hamming Window, which provided a 43 Hz frequency resolution and 56 Hz bandwidth resolution on the measurements. We measured the following parameters using the automatic parameter measurement function: minimum frequency, maximum frequency, and peak frequency. Automatic parameter measurements were used to reduce bias and increase consistency of the measures (Zollinger et al. 2012; Ríos-Chelén et al. 2017). Bandwidth (frequency difference) was calculated as the difference between the maximum and minimum frequency. We analyzed at least three vocalizations for each individual included in this study (range: 3–63). All vocalizations were measured separately and then averaged for each individual.

For the automatic parameter measurements, a -15 dB threshold and 25 ms hold time was set, with the measurement taken at the start, center and the end of the vocalization. The cut-off frequency function was used on the recordings before measures were taken, based on the visual inspection of the spectrogram; a high pass frequency filter removed low-frequency noise, and a low pass filter was applied on those recordings which contained continuous high-frequency noise such as insect sounds. Other noise that could potentially affect the automatic measurement was cleared using the standard eraser cursor function in Avisoft using manual visual judgment. We did not include any recordings where songs were so heavily masked that the vocalization could not be clearly distinguished.

From the same recordings, the maximum frequency of the ambient noise (excluding insect sounds) and the minimum frequency of insect sounds were measured with automatic measurements set at the same setting for analyzing bird vocalizations. High-frequency insect noise is mainly produced by cicadas, and there are twenty known Cicadidae species recorded in Hong Kong (Hong Kong Entomological Society 2014), but we could not identify the exact species in each recording.

### Statistical Analysis

All statistical analyses were run in R v3.5.1 (R Core Team 2018) and figures were produced in ggplot2 (Wickham 2016).

We first tested the effects of the following variables; 1) species, 2) location (urban, peri-urban), 3) cicada (present or not), as fixed factors; and 4) noise level as a covariate, in a linear model (lm) with each of the song response parameters (minimum frequency, maximum frequency, peak frequency, and bandwidth. However, species were significantly different in their responses, as expected, and there were three way interactions with species, location, and cicada. Therefore, we ran separate linear models (using the package lme4; Bates et al. 2015) for the response variables for each species each including, 1) location, and 2) cicada, as fixed factors, and 3) the covariate noise Level (dB).

As the factors in the model were not balanced we used Type III tests with contrasts and used the drop1 for all models. All models included the effects of location and cicada, including the interaction between the two, and noise level, as there is strong evidence that these factors are likely to affect song parameters. However, we also tested whether including a location × noise level interaction term significantly improved model performance (using Anova comparison of models), if not the term was removed. Tukey post hoc comparisons were conducted using lsmeans (Lenth 2016) to identify significant differences in location/cicada.

We verified that final models satisfied regression model assumptions by examining residual plots.

A second model to test for the effect of minimum cicada noise frequency was run for each species and each vocal parameter including the following factors, 1) location, 2) noise level, and 3) cicada minimum frequency.

**Figure 3:**
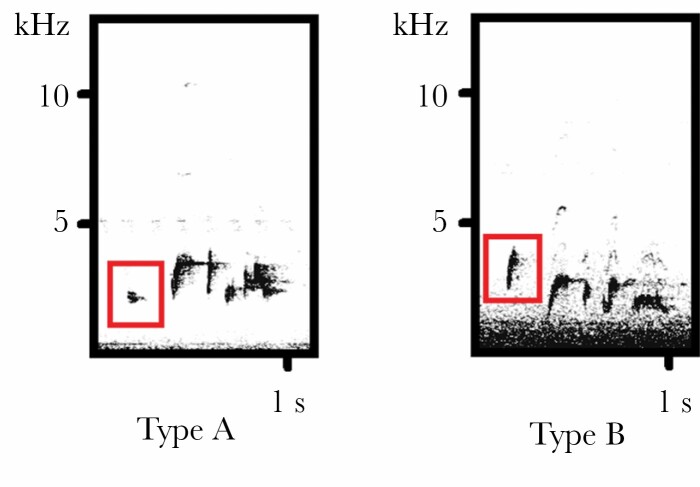
Two different types of Red-whiskered Bulbul song, grouped based on the structure of the first note (highlighted in red).

In Red-whiskered Bulbul songs, we observed two acoustic phenotypes based on differences in the first note (Figure 3). In “Type A” songs, the first note is a lower frequency syllable (minimum frequency, mean = 1.6 kHz, range 1.4–1.9 kHz; maximum frequency, mean=3.3 kHz, range 2.9–3.7 kHz), while in “Type B” songs, the first note is a higher frequency syllable (minimum frequency, mean = 1.7 kHz, range 1.5–2.3 kHz; maximum frequency, mean=3.5 kHz, range 3–4.3 kHz). We tested for an effect on which note type was used in a linear model with a binomial error distribution including the factors; 1) location, 2) cicada, and 3) noise level. A second model was run as above including cicada minimum frequency instead of cicada presence absence.

Significance of the model coefficients are given in the Supplementary Table S1.

## RESULTS

### Ambient noise levels

The background noise level in urban sites (mean ± SE = 75.23 ± 0.82 dB(C); minimum = 70.96 dB(C); maximum = 80.01 dB(C); *N* = 11) was significantly higher (Independent t test: *t*_20_ = 5.631, *P* < 0.001) than in peri-urban sites (mean ± SE = 69.31 ± 0.65 dB(C); minimum = 64.23 dB(C); maximum = 71.85 dB(C); *N* = 11). The maximum frequency of anthropogenic noise in urban sites (mean ± SE = 1.425 ± 0.038 kHz; minimum = 0.667 kHz; maximum = 2.569 kHz; *N* = 137) was also significantly higher (independent t test: *t*_270_ = −13.773, *P* = <0.001) than in peri-urban sites (mean ± SE = 0.749 ± 0.031 kHz; minimum = 0.200 kHz; maximum = 1.835 kHz; *N* = 135).

The minimum frequency of cicada sounds was quite variable in frequency within both urban (mean ± SE = 4.889 ± 0.094 kHz; minimum = 2.842 kHz; maximum = 7.091 kHz; *N* = 74) and peri-urban sites (mean ± SE = 5.199 ± 0.092 kHz; minimum = 2.282 kHz; maximum = 8.182 kHz; *N* = 82). The average cicada noise frequency was significantly higher in peri-urban area (independent t test: *t*_154_ = −2.359, *P* = 0.020).

### Swinhoe’s white-eye

**Figure 4:**
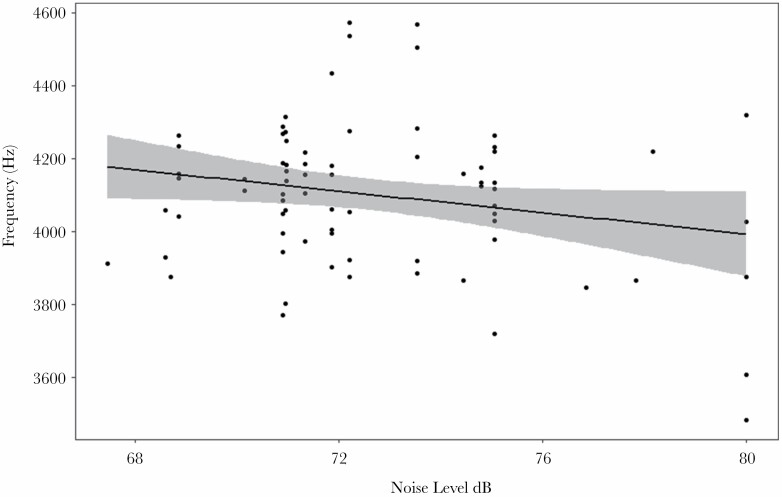
Scatterplot showing the relationship between the minimum frequency of the Swinhoe’s White-eye and noise level, including the 95% confidence intervals for the fitted line

There was a negative relationship between the minimum frequency of Swinhoe’s White-eye vocalizations and noise level (*F*_1,75_ = 6.39, *P* = 0.014, Figure 4, Supplementary Table S1), when controlling for location. No other vocal parameters were significantly affected by the noise conditions. There was also no effect of cicada minimum frequency on any of the song parameters.

### Eurasian tree sparrow

**Figure 5:**
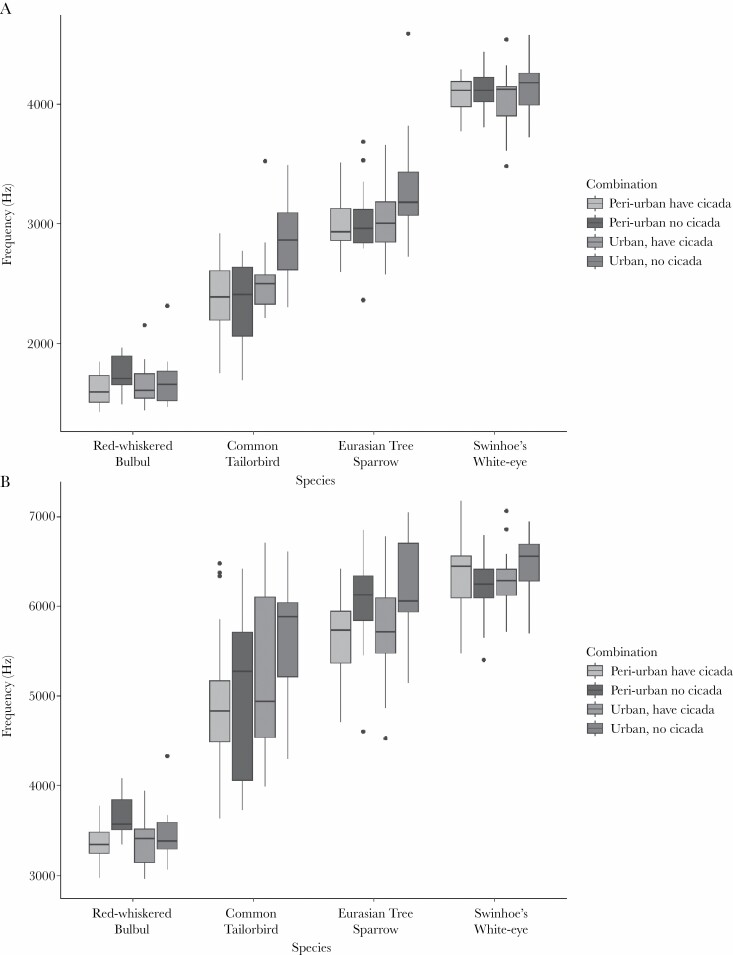

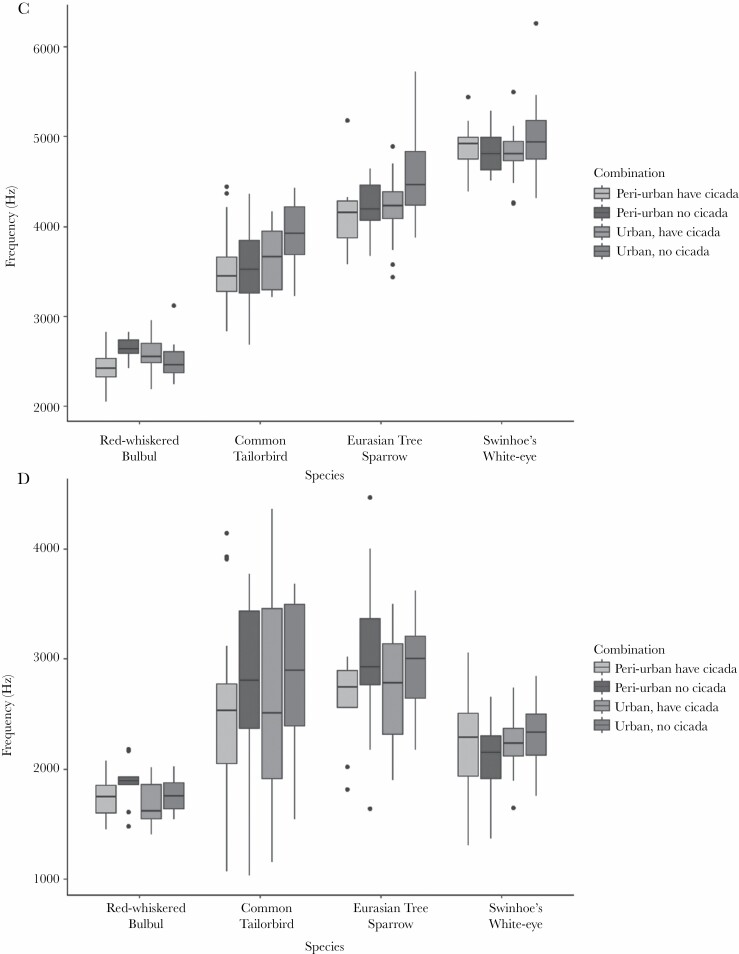
Boxplots showed the mean value of the four acoustic parameters (a) minimum frequency, (b) maximum frequency, (c) peak frequency, and (d) bandwidth, in the four different situations peri-urban, no insect noise; peri-urban, with insect noise; urban, no insect noise; urban, with insect noise of all four species studied.

Minimum frequency was higher in urban areas, (*F*_1,73_ = 5.82, *P* = 0.018, Figure 5a, Table 2) mainly due to the fact that birds in urban areas raised their minimum frequency when no cicada were present (Tukey’s *P* < 0.05).

**Table 2:** Mean value of acoustic parameters of the four bird species studied in the four different ambient noise environments (peri-urban, no insect noiseperi-urban, with insect noise; urban, no insect noise; urban, with insect noise)

Average Frequency ± SE (Hz)				
	Peri-urban		Urban	
	No insect noise	With insect noise	No insect noise	With insect noise
Swinhoe’s White-eye (*Zosterops simplex*)				
*N*	19	20	22	19
Minimum Frequency	4120 ± 45	4084 ± 44	4152 ± 42	4041 ± 45
Maximum Frequency	6229 ± 89	6300 ± 87	6457 ± 83	6270 ± 89
Peak Frequency	4838 ± 70	4885 ± 68	4976 ± 65	4814 ± 70
Bandwidth	2109 ± 80	2216 ± 79	2305 ± 74	2229 ± 80
Eurasian Tree Sparrow (*Passer montanus*)				
*N*	16	10	22	30
Minimum Frequency	3020 ± 81	2989 ± 102	3273 ± 69	2999 ± 59
Maximum Frequency	6064 ± 130	5616 ± 164	6220 ± 110	5703 ± 94
Peak Frequency	4237 ± 90	4135 ± 114	4525 ± 77	4222 ± 66
Bandwidth	3044 ± 126	2627 ± 160	2947 ± 108	2704 ± 92
Common Tailorbird (*Orthotomus sutorius*)				
*N*	9	32	10	12
Minimum Frequency	2320 ± 109	2392 ± 58	2863 ± 103	2553 ± 95
Maximum Frequency	5022 ± 262	4900 ± 139	5680 ± 249	5196 ± 227
Peak Frequency	3509 ± 141	3486 ± 75	3897 ± 134	3646 ± 122
Bandwidth	2702 ± 269	2508 ± 142	2818 ± 255	2642 ± 233
Red-whiskered Bulbul (*Pycnonotus jocosus*)				
*N*	9	20	9	13
Minimum Frequency	1758 ± 60	1625 ± 40	1705 ± 60	1680 ± 50
Maximum Frequency	3639 ± 88	3362 ± 59	3477 ± 88	3382 ± 73
Peak Frequency	2649 ± 65	2445 ± 44	2526 ± 65	2551 ± 54
Bandwidth	1881 ± 64	1736 ± 43	1773 ± 64	1701 ± 53

Maximum and peak frequency were both significantly lower when cicadas were present (Max frequency: *F*_1,73_ = 14.22, *P* = 0.0003, Figure 5b; Peak frequency: *F*_1,73_ = 5.14, *P* = 0.026, Figure 5c; Table 2). The reduction in maximum and peak frequency in response to cicadas was significant only in the urban areas (Tukey’s *P* < 0.05). Bandwidth was also narrower in the presence of cicadas (*F*_1,73_ = 7.23, *P* = 0.009, Figure 5d, Table 2).

There was no effect of cicada minimum frequency on any of the song parameters.

### Common tailorbird

There was a significant effect of location, and an interaction between location and cicada presence, on minimum frequency (Location: *F*_1,58_ = 6.04, *P* = 0.017; Location × Cicada: *F*_1,58_ = 4.28, *P* = 0.043; Figure 5a; Table 2). Posthoc tests showed that minimum frequency was lower in peri-urban areas, both with and without cicada, compared to urban areas when cicada were absent (Tukey’s *P* < 0.05).

There were significant interactions between noise and location for both maximum frequency and bandwidth; *n* increase in noise level led to an increase in both maximum frequency and bandwidth in peri-urban areas, but an increase in noise level in urban areas led to a decrease in both parameters (Maximum frequency: *F*_1,57_ = 4.33, *P* = 0.042, Figure 5b; Bandwidth, *F*_1,57_ = 8.26, *P* = 0.006, Figure 5d; Table 2).

**Figure 6:**
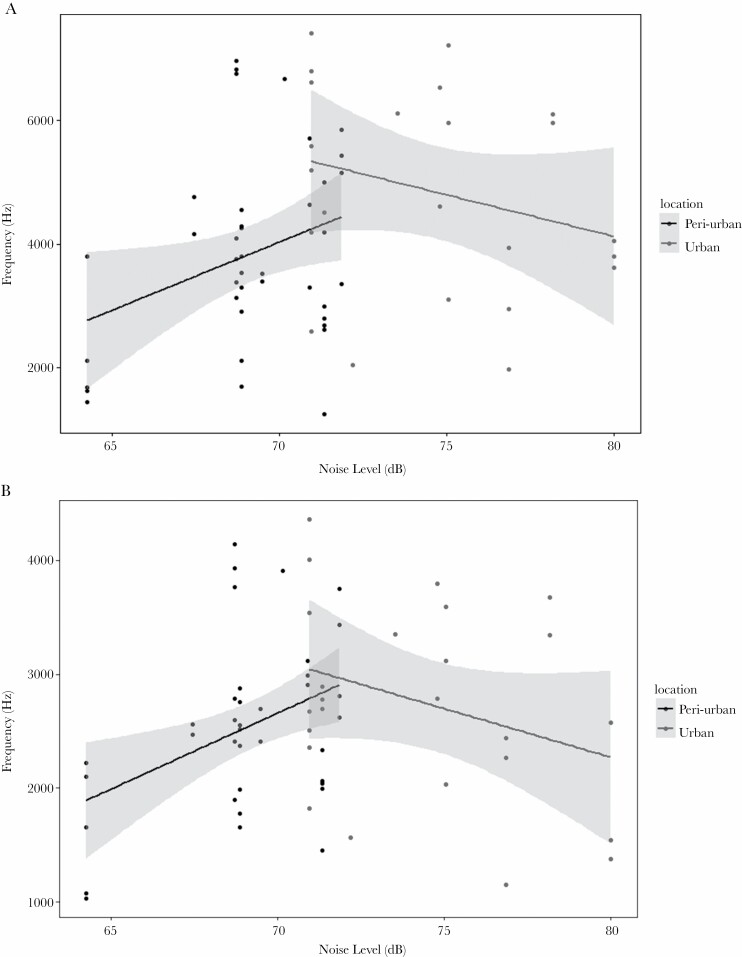
Scatterplot showing the relationship between the a) maximum and b) bandwidth frequency, of the Common Tailorbird and noise level separately for urban and peri-urban areas, including the 95% confidence intervals for the fitted lines

There was also a main effect of location on both maximum frequency and bandwidth (Maximum frequency: *F*_1,57_ = 4.57, *P* = 0.037, Figure 5b; Bandwidth, *F*_1,57_ = 8.26, *P* = 0.006; Figure 5d; Table 2). Maximum frequency (Figure 6a) and bandwidth (Figure 6b) both increased in urban areas.

Cicada frequency had no effect on any of the song parameters.

### Red-whiskered bulbul

The maximum frequency was lower across both locations when there were cicadas present (*F*_1,46_ = 5.69, *P* = 0.021, Figure 5b, Table 2). None of the other song frequency characteristics were significantly affected by environmental variables, including cicada minimum frequency.

Note type was affected by location (Chisq_1,46_ = 12.93, *P* = 0.0003) with note type B being produced much more frequently in urban areas (Table 3). The difference in maximum frequency between note type A and B was significant, but relatively low mean difference = 173 Hz, *t*_49_ = 2.23, *P* = 0.03)

**Table 3:** Observed frequency of Red-whiskered Bulbul song type in different noisy conditions

Song type	Peri-urban	Urban
Type A	16	2
Type B	13	20

## Discussion

We observed, generally, that the species included in this study increased the frequency of their vocalizations in urban areas with low-frequency background noise, and reduced the frequency in the presence of high-frequency cicada noise, as found in previous studies (Dingle et al. 2008; Kirschel et al. 2009; Hu and Cardoso 2010; Slabbekoorn 2013; Lenske and La 2014; Roca et al. 2016). However, the presence of both low-frequency anthropogenic noise and high-frequency cicada sounds affected the bird vocalizations in a complex manner, causing some species to sing differently compared to when exposed to only one type of noise.

Common Tailorbirds and Eurasian Tree Sparrows both sang with higher minimum frequencies in urban areas, but only when cicadas were not present. In urban areas, when exposed to both low-frequency anthropogenic noise and high-frequency cicada noise, vocalization frequencies did not differ from those of birds in peri-urban sites. Urban Eurasian Tree Sparrows also decreased the maximum and peak frequencies of their songs, leading to lower bandwidths, when cicada noise was present, in addition to the elevated levels of anthropogenic noise. The narrow frequency window available to these two species for avoiding signal masking thus appears to lead to reduced ability to make signal adjustments to avoid masking when it is required for avoiding both high and low-frequency masking noise. The minimum frequency of these two species ranges from 2 to 3 kHz, so small upwards shifts in minimum frequency could lead to increased masking by cicada choruses. Any benefit gained from increased frequencies in response to anthropogenic noise would be counteracted by the costs of signal masking by cicadas, leading to no net benefit in making vocalization adjustments. There could even be an additional cost of trying to squeeze the song from the top and the bottom as low bandwidth songs may be less attractive signals.

In Common Tailorbirds, we also found that the response to increasing noise levels differed between urban and peri-urban sites; increasing noise levels in peri-urban areas led to an increase in maximum frequency and bandwidth, but a decrease in these two parameters in urban areas. Given that the noise was, in general, much louder in the urban sites, a potential explanation for this is that when noise reaches a certain threshold, birds are constrained in some way from making any further adjustments to additional increases in noise levels. In this case, birds may eventually appear to reach a maximum increase in frequency and then either stop shifting upward or even begin to decrease in frequency (Hu and Cardoso 2010; Shiba et al. 2015; Guo et al. 2016).

The vocalizations of Swinhoe’s White-eye, which had the highest frequencies of the four species studied, did not differ between urban and peri-urban populations, although we found a negative relationship between background noise levels and minimum frequency, contrary to predictions. The minimum frequency of this species’ vocalizations is 4 kHz, much higher than the peak frequency of anthropogenic noise and thus would be unlikely to suffer from signal masking from anthropogenic noise, potentially explaining why they did not increase the minimum frequency of their songs in response to urban noise (Parris and Schneider 2009; Hu and Cardoso 2010; Parris and McCarthy 2013; Lowry et al. 2019). In fact, we found the opposite response: minimum frequency of their vocalizations decreased with increasing noise levels (which we discuss in more detail below). Given the frequency range of the Swinhoe’s White-eye vocalizations (4 - 6.5 kHz), this species would have the highest amount of overlap with, and thus a high potential for masking by, the frequency of the cicada vocalizations. However, we did not find any significant impact of cicada sounds in this species. Due to the extensive overlap between the Swinhoe’s White-eye vocalizations and the cicada noise, Swinhoe’s White-eyes may simply be unable to sing in a frequency that would completely avoid masking.

Red-whiskered Bulbul vocalizations, which have the lowest minimum frequency among the four species included in this study (1.4–1.6 kHz), were predicted to have the strongest response to noise, due to the overlap with anthropogenic noise (Parris and McCarthy 2013). However, our results showed that songs of this species did not differ between urban and peri-urban areas or with increasing anthropogenic noise levels. However, we did find that birds in urban areas sang Type B songs more frequently—the first note in this song type had a higher frequency than in Type A songs. So rather than shifting the minimum frequency of the whole song, the bulbuls appear to avoid signal overlap by replacing a low-frequency syllable with a high-frequency syllable in the introductory note, as in Great Tits and Northern Mockingbirds (*Mimus polyglottos)* (Slabbekoorn 2013; Walters et al. 2019). In addition, we found an impact of cicada noise on their songs; the Red-whiskered Bulbuls sang with lower maximum frequencies in the presence of cicadas. These results mirror results from other species that sing with lower maximum frequencies in the presence cicada noises (Gray-breasted Wood-wrens, *Henicorhina leucophrys*, Dingle et al. 2008; Green Hylia *Hylia prasina*, Kirschel et al. 2009). White-crowned Sparrow (*Zonotrichia albicolis*) reduced song bandwidth during frog chorusing (Lenske and La 2014).

It has been argued that observed increases in frequency in noisy areas is simply an involuntary byproduct of birds singing at a higher amplitude (the “Lombard effect,” Nemeth et al. 2013). While we did not measure song amplitude in this study, we found evidence in all four species that frequencies decreased in the presence of background noise, which would seem to contradict this hypothesis. In Swinhoe’s White-eyes, minimum frequencies decreased in areas with higher levels of anthropogenic noise. For urban Eurasian Sparrows, maximum and peak frequencies, along with bandwidth, declined in the presence of cicadas. Finally, for the Common Tailorbirds, increased noise levels led to a decrease in maximum frequency and bandwidth in urban areas (in contrast to the impact of increasing anthropogenic noise levels in peri-urban areas which led to an increase in these parameters). These results provide a counterpoint to the argument that birds simply respond to noise by increasing the amplitude of their songs, leading to an involuntary increase in frequencies and suggest that birds can control the frequency and amplitude of their vocalizations independently.

Frequency characteristics of biotic noises in our study were more variable than low-frequency anthropogenic noise, likely due to the diversity of species of cicada; at least twenty species of cicada have been recorded in Hong Kong (Hong Kong Entomological Society 2014). Although there is limited information available on the frequency range of each of these species, it is likely that there is significant variation in the frequency, amplitude, and timing of calls between these species. In our study, the minimum frequency of cicada choruses ranged from 2 kHz up to 8 kHz, with a mean value of 5 kHz. This variation could imply that the impact of bird songs will be highly variable, depending on which cicada species is present and on the frequency range of the birds.

Overall our results together imply that a more complex soundscape, including both urban and biotic noise, may limit potential song adaptation as well as our ability to predict how birds respond in such complex situation. Response to noise/urbanization appeared to be reversed in urban areas when cicadas were also present. Previous investigations into the impact of different types of noise have indicated responses differ. When Silvereyes (*Zosterops lateralis)* were experimentally exposed to both low and high-frequency noise, they lowered the minimum frequency of their vocalizations when exposed to high-frequency noise, but showed only a small effect in response to low-frequency noise (Potvin and Mulder 2013). White-throated Sparrows had different vocalization adjustments in response to biotic and abiotic noise separately (Lenske and La 2014). However, unlike in our study, neither study compared the effect of both sources simultaneously on bird vocalizations. LaZerte et al. (2016) showed the response of Black-capped Chickadees (*Poecile atricapillus*) to a noise varies between quiet and noisy areas; males in noisy environments shifted their frequency upward in response to increasing levels of background noise, but shifted downwards in quiet areas. These examples, in addition to our results, suggest that there may be potential trade-offs in responding to high and low-frequency noise-sources simultaneously. It is also possible that under scenarios when it becomes impossible to adjust frequency parameters to avoid song masking, birds might adjust other parameters to compensate.

Temporal song characteristics, amplitude, and the timing of vocalizations have all been shown to play a role in a species’ response to urban noise (Luther 2009; Slabbekoorn 2013; Lenske and La 2014; Hart et al. 2015; Stanley et al. 2016; Zollinger 2017). As we did not measure these aspects of the vocalizations in this study, it is possible that changes in these features may explain some of the absence of frequency response observed in our study.

It is notable that insects could be affected by anthropogenic noise themselves, and have a similar response to birds such as altering their acoustic signals (Costello and Symes 2014, Morley et al. 2014). As a result, there could be a cumulative effect as insects adjust their acoustic signal in response to urban noise, and then birds respond to both noises (Kirschel et al. 2009). The nature of both anthropogenic noise and insect sounds could vary from time to time, and place to place. Studies showed birds change their song based on the noise profile at the time they sing, rather than the overall noise level (Shannon et al. 2016, Gentry et al. 2017). Hence, birds may need to flexibly adjust their vocalizations based on the real-time situation. However, these adjustments cannot always maintain the original communication function fully under anthropogenic noise (Moiron et al. 2015; Luther et al. 2016). With the addition of intense insect noise, communication effectiveness may be further reduced. Birds that live in cities with intensive biotic noise, such as tropical and subtropical cities, are facing heavier pressure on communication than was previously known. These birds might struggle to communicate under the influence of both noises.

## Conclusions

The results of our study complement the growing body of evidence that birds adjust the frequencies of their vocalizations in response to anthropogenic noise, but highlight that this response is not straightforward when multiple noise sources are present. The response of the fours species included in this study to background noise differed depending on the frequency of the noise source, and differed when in the presence of both high and low-frequency noise sources simultaneously. The presence of two different noise sources may therefore present a trade-off between increasing and decreasing frequency characteristics in order to avoid signal masking.

As low-frequency anthropogenic noise has already been shown to reproductive success and fitness in urban birds (Brumm and Slabbekoorn 2005; Slabbekoorn 2013; McMullen et al. 2014; Moiron et al. 2015; Shannon et al. 2016; Kleist et al. 2018), it seems likely that birds in cities simultaneously exposed to high and low-frequency noise will face additional challenges. As these areas are very likely to be in tropical cities where little attention has been paid to the impact of noise on birds, we strongly encourage more studies to understand how birds adjust their songs when exposed to multiple noise source types and the impact on reproduction and survival.

## Supplementary Material

arab058_suppl_Supplementary_Figure-1

arab058_suppl_Supplementary_Figure-2

arab058_suppl_Supplementary_Figure-3

arab058_suppl_Supplementary_Figure-4

arab058_suppl_Supplementary-Table-S1

## Data Availability

Analyses reported in this article can be reproduced using the data provided by To et al. 2021.
