# Supplementary figures and images for: Multiple constraints on urban bird communication: both abiotic and biotic noise shape songs in cities
Source: Behav Ecol. 2021 Jul 13;32(5):1042–53. doi: 10.1093/beheco/arab058 (PMC8528541; doi:10.1093/beheco/arab058)

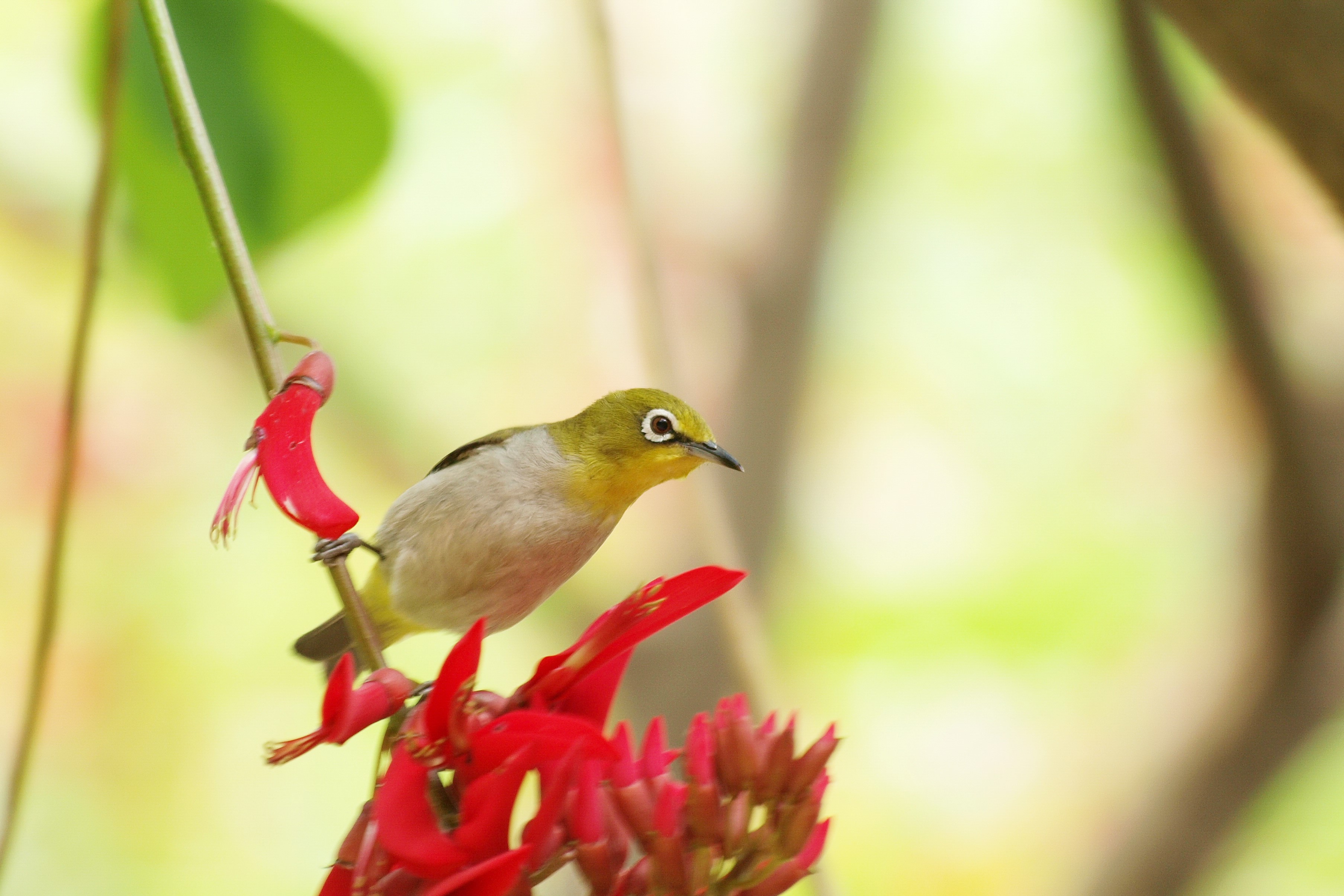

Supplement: arab058_suppl_Supplementary_Figure-1 [file arab058_suppl_supplementary_figure-1.jpeg]

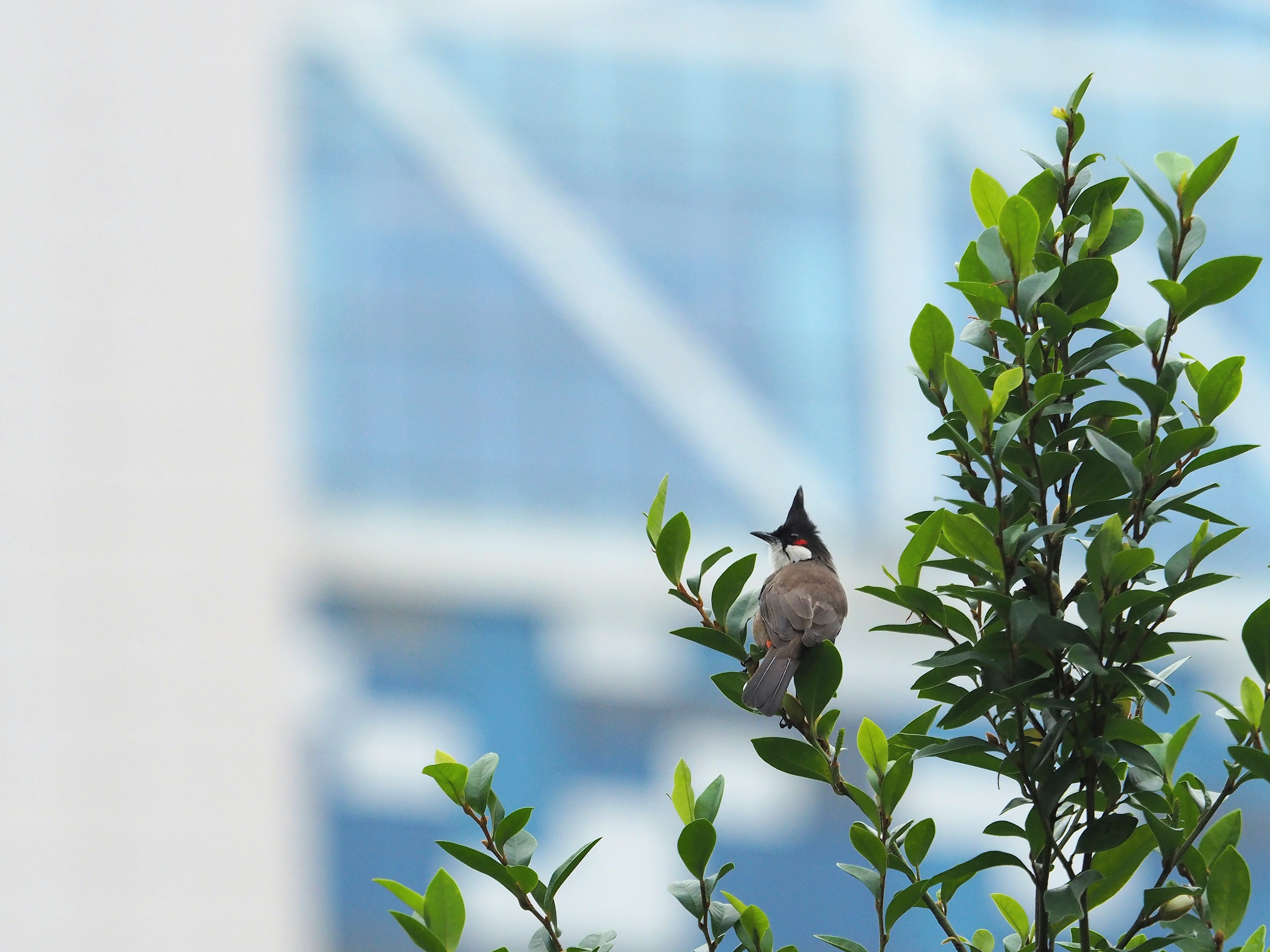

Supplement: arab058_suppl_Supplementary_Figure-2 [file arab058_suppl_supplementary_figure-2.jpeg]

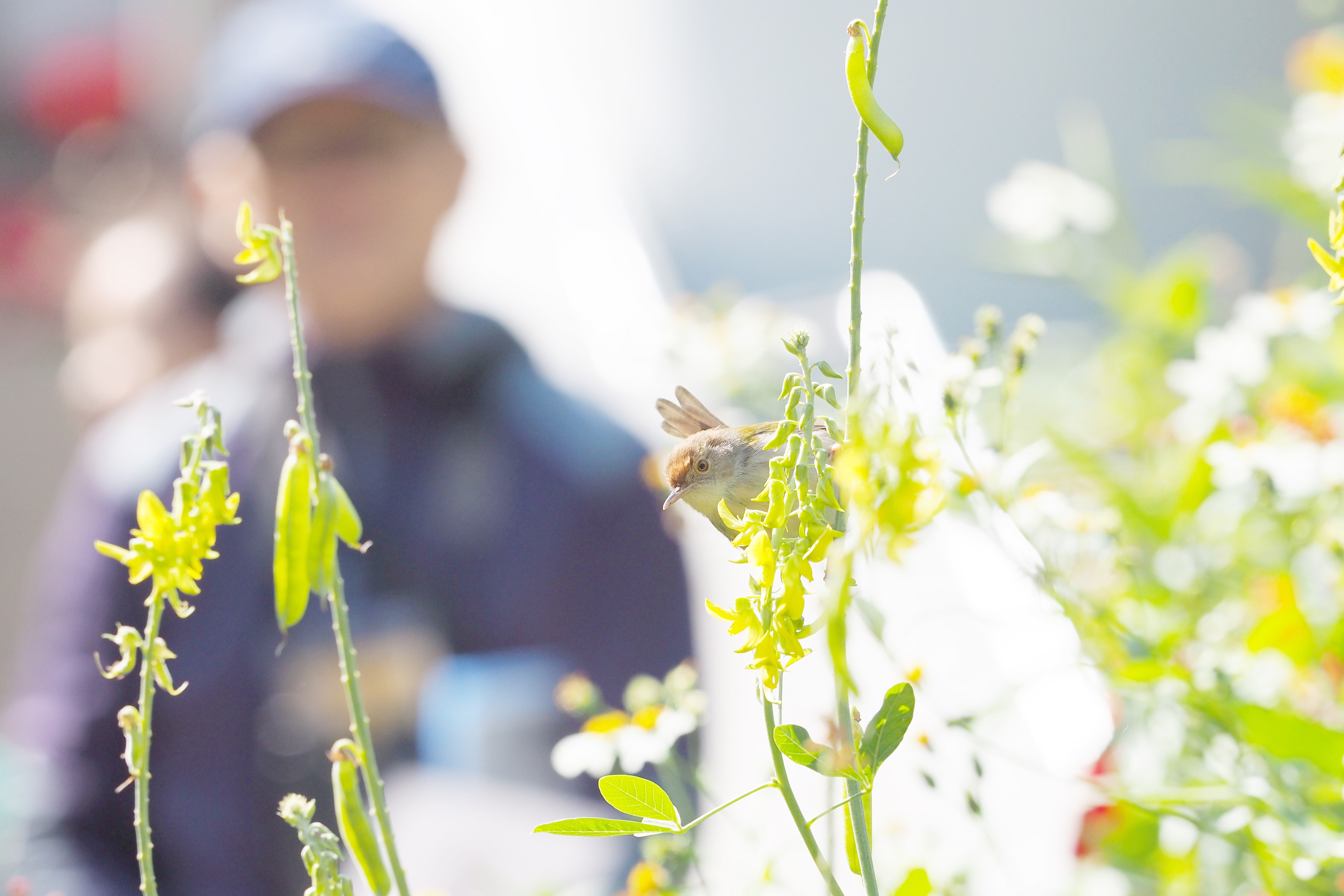

Supplement: arab058_suppl_Supplementary_Figure-3 [file arab058_suppl_supplementary_figure-3.jpeg]

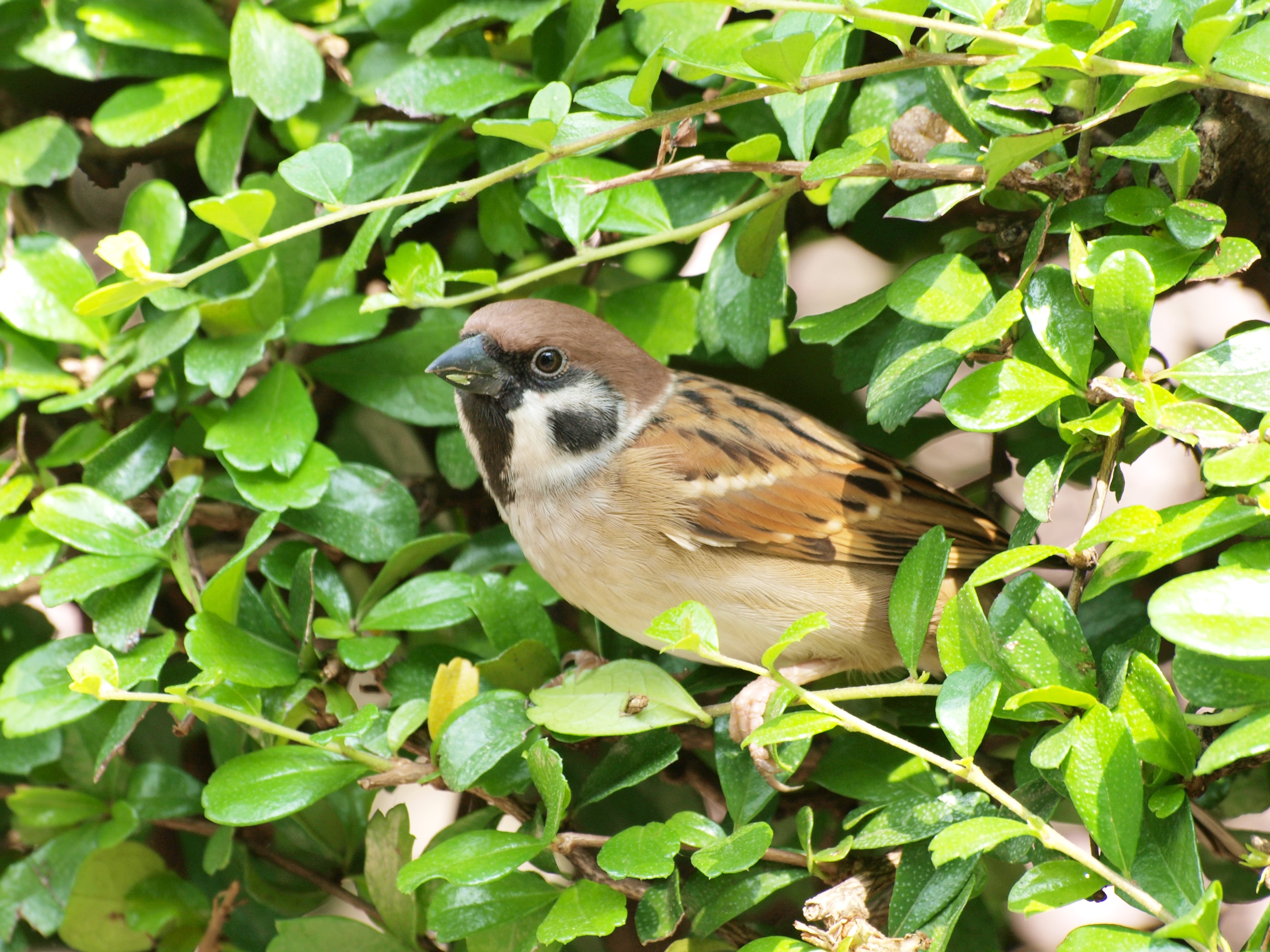

Supplement: arab058_suppl_Supplementary_Figure-4 [file arab058_suppl_supplementary_figure-4.jpeg]
