# Supplementary material for: Multiple constraints on urban bird communication: both abiotic and biotic noise shape songs in cities
Source: Behav Ecol. 2021 Jul 13;32(5):1042–53. doi: 10.1093/beheco/arab058 (PMC8528541; doi:10.1093/beheco/arab058)
Supplement: arab058_suppl_Supplementary-Table-S1 [file arab058_suppl_supplementary-table-s1.docx]

Table S1. Estimates for coefficients of linear models for significant results

| **Song trait** | **Species** | **Variable** | **Estimate** | ***t* (df)** | ***P*** |
| --- | --- | --- | --- | --- | --- |
| Minimum Frequency | Swinhoe’s White eye | Noise Level | 30.05±11.89 | 2.53 (75) | 0.014 |
|  | Eurasian Tree Sparrow | Location | 183.38±76.01 | 2.41 (73) | 0.018 |
|  | Common Tailorbird | Location | 156.95±63.86 | 2.46 (58) | 0.017 |
|  |  | Location*Cicada | 99.82±48.26 | 2.06 (58) | 0.043 |
| Maximum Frequency | Eurasian Tree sparrow | Cicada | 241.28±63.99 | 3.77 (73) | 0.0003 |
|  | Common Tailorbird | Location | 5830.66±2727.40 | 12.14 (57) | 0.037 |
|  |  | Location*Noise evel | 78.77±37.84 | 2.08 (57) | 0.042 |
|  | Red-whiskered Bulbul | Cicada | 94.07±39.45 | 2.38 (46) | 0.021 |
| Peak Frequency | Eurasian Tree Sparrow | Cicada | 100.83±44.47 | 2.267 (73) | 0.026 |
| Bandwidth | Eurasian Tree Sparrow | Cicada | 166.7±61.98 | 2.69 (73) | 0.009 |
|  | Common Tailorbird | Location | -7794.86±2715.08 | 2.87 (57) | 0.006 |
|  |  | Location*Noise Level | 108.241±37.67 | 2.87 (57) | 0.006 |
